# The Role of ApoE Expression and Variability of Its Glycosylation in Human Reproductive Health in the Light of Current Information

**DOI:** 10.3390/ijms22137197

**Published:** 2021-07-04

**Authors:** Monika Kacperczyk, Agnieszka Kmieciak, Ewa Maria Kratz

**Affiliations:** Department of Laboratory Diagnostics, Division of Laboratory Diagnostics, Faculty of Pharmacy, Wroclaw Medical University, Borowska Street 211A, 50-556 Wroclaw, Poland; monika.kacperczyk@umed.wroc.pl (M.K.); agnieszka.kmieciak@umed.wroc.pl (A.K.)

**Keywords:** apolipoprotein E, ApoE concentration, ApoE glycosylation, human fertility, reproductive tract disorders

## Abstract

Apolipoprotein E (ApoE), a 34-kDa glycoprotein, as part of the high-density lipoprotein (HDL), has antioxidant, anti-inflammatory and antiatherogenic properties. The variability of ApoE expression in the course of some female fertility disorders (endometriosis, POCS), and other gynecological pathologies such as breast cancer, choriocarcinoma, endometrial adenocarcinoma/hyperplasia and ovarian cancer confirm the multidirectional biological function of ApoE, but the mechanisms of its action are not fully understood. It is also worth taking a closer look at the associations between ApoE expression, the type of its genotype and male fertility disorders. Another important issue is the variability of ApoE glycosylation. It is documented that the profile and degree of ApoE glycosylation varies depending on where it occurs, the type of body fluid and the place of its synthesis in the human body. Alterations in ApoE glycosylation have been observed in the course of diseases such as preeclampsia or breast cancer, but little is known about the characteristics of ApoE glycans analyzed in human seminal and blood serum/plasma in the context of male reproductive health. A deeper analysis of ApoE glycosylation in the context of female and male fertility will both enable us to broaden our knowledge of the biochemical and cellular mechanisms in which glycans participate, having a direct or indirect relationship with the fertilization process, and also give us a chance of contributing to the enrichment of the diagnostic panel in infertile women and men, which is particularly important in procedures involved in assisted reproductive techniques. Moreover, understanding the mechanisms of glycoprotein glycosylation related to the course of various diseases and conditions, including infertility, and the interactions between glycans and their specific ligands may provide us with an opportunity to interfere with their course and thus develop new therapeutic strategies. This brief overview details some of the recent advances, mainly from the last decade, in understanding the associations between ApoE expression and some female and male fertility problems, as well as selected female gynecological diseases and male reproductive tract disorders. We were also interested in how ApoE glycosylation changes influence biological processes in the human body, with special attention to human fertility.

## 1. Introduction

Apolipoprotein E (ApoE) is a 34-kDa glycoprotein. The primary translation product consists of 317 amino acids and includes an 18-amino acid signal peptide [1], and a mature ApoE is composed of 299 amino acids [2]. Physiologically, ApoE does not cross the blood–brain barrier (BBB), but is present both in the periphery and in the central nervous system (CNS) [3]. Around the perimeter, ApoE is secreted from many cells throughout the human body, such as hepatic parenchymal cells, monocytes, macrophages, adipocytes and muscle cells [4]. In the CNS the astrocytes, vascular mural cells, pericytes, oligodendrocytes and choroid plexus are the main suppliers of ApoE [5,6]. Peripheric ApoE and the ApoE of CNS differ in their structure [7] and the CNS ApoE is more highly glycosylated on the C-terminal part of the protein chain, which has a large impact on its function [8].

**Figure 1:**
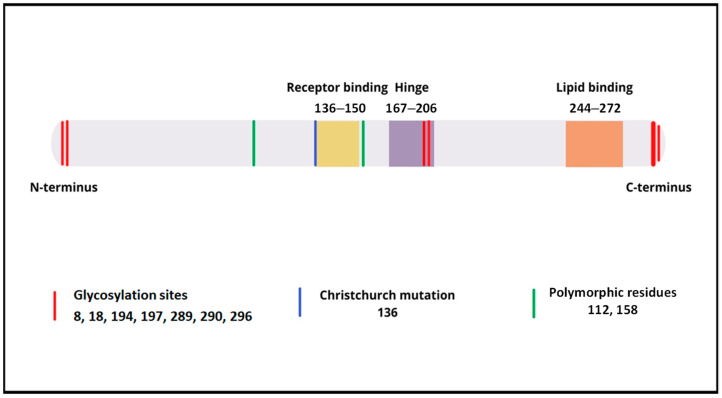
Schematic structure of ApoE. Possible O-glycosylation sites: Thr8, Thr18, Thr194, Ser197, Thr289, Ser290 and Ser296 [7]. The Christchurch mutation results in a Arg136–Ser substitution [18]. Cys and/or Arg may be present at the 112th and 158th position in the amino acid chain, which determines the occurrence of the respective isoforms of ApoE [11,15]. Modification based on Liu et al. [22].

ApoE consists of two primary domains linked by a flexible loop region, each characterized by a distinct structure and function [1,2] (see Figure 1). The N-terminal domain, including a four antiparallel helix bundle, comprises the receptor-binding region (136–150 aa) and the heparan sulphate proteoglycans (HSPGs) binding region [9]. The C-terminal domain consists of amphipathic α-helices, the high affinity lipid-binding region (244–272 aa), and the region responsible for ApoE self-association (267–299 aa) [10]. ApoE is coded by gene 19q13.32 on the long arm of chromosome 19 [11,12], and the *ApoE* gene is associated with another apolipoprotein gene, *ApoC-I* [13]. Human *ApoE* is characterized by its polymorphic nature and three allelic variants occur in the gene of this protein at the single gene locus, namely ɛ2, ɛ3 and ɛ4 [14]. They encode the ApoE isoforms E2, E3 and E4, respectively. Between the isoforms differences were found in amino acid substitutions in the 112 and 158 positions [15]. ApoE2 possesses a cysteine at both positions, ApoE3 possesses a cysteine at 112, but an arginine at 158, and ApoE4 possesses an arginine at both positions [11]. ApoE3 is the most common isoform, occurring in 70–80% of the human population [16]. ApoE1, ApoE5 and ApoE7 are rare isoforms, which have different variants of amino acid substitution [17]. Additionally, a number of point mutations of ApoE are observed, notable among which is the Christchurch mutation. In this rare variant of ApoE2, a substitution of Arg136–Ser occurs [18]. The Christchurch mutation may contribute to the pathogenesis of type III hyperlipoproteinemia [19], and probably plays an as yet unclear role in the development of Alzheimer’s disease (AD) [20,21].

This short review details some of the recent advances, mainly from the last ten years, focusing on the question of how ApoE and its glycosylation changes influence biological processes in the human body, especially with regard to human fertility and reproductive health disorders. Taking into account that the above topics are not very deeply explored, they may constitute an interesting aim for future research and open new opportunities for the explanation of some of the molecular mechanisms of ApoE action.

## 2. ApoE Expression in Selected Female and Male Diseases

**Figure 2:**
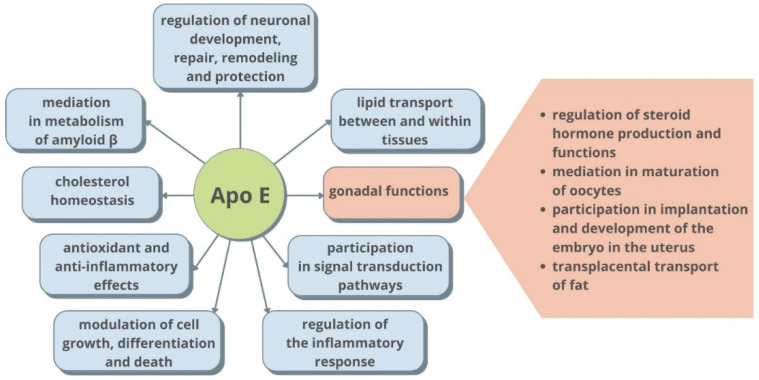
Main functions of ApoE in the human body.

The importance of ApoE concentration in clinical practice is still the subject of numerous studies. In both the cerebrospinal fluid (CSF) and blood plasma, the concentration of ApoE is strongly isoform-dependent [23]. The level of ApoE is lower in individuals that possess ApoE4 and higher in subjects with an ApoE2 isoform [24,25]. Moreover, the concentration of blood plasma ApoE is also affected by the concentration of triglyceride-rich lipoproteins, age and sex [26]. Physiologically ApoE concentration is 40–80 µg/mL in blood plasma and 3–5 µg/mL in CSF [27]. In light of the information reported by various authors, based on the results of their studies, there is a clear need for further research on the usefulness of ApoE concentration determinations in many diseases, in the context of the possible diagnostic and/or therapeutic usefulness of this parameter. The main ApoE functions in the human body, including those directly associated with the maintenance or disorders of reproductive health, are shown in Figure 2. Table 1 gathers the main information about the association of ApoE expression with other gynecological and male reproductive tract diseases.

### 2.1. Reproductive System Disorders

#### 2.1.1. ApoE in Female Fertility

ApoE is involved in the physiological functions of the female gonads [28]. In 1996, Gerdes et al. [29] already hypothesized that the ApoE genotype could also affect reproductive efficiency, taking into account that ApoE is involved in gonadal function, implantation of the embryo in the uterus, and transplacental transportation of fat. This glycoprotein stimulates the production of androgens by theca cells, which indirectly affects the production of estrogen, determining the proper maturation of follicles [30]. A high concentration of ApoE is responsible for the inhibition of androgen production, while a low concentration stimulates it [30]. The involvement of lipoproteins and sterols in the regulation of ovarian function is complex due to the multitude of cell types and compartments in ovarian follicles. ApoE is implicated in cholesterol transport within ovarian follicles to regulate steroidogenesis, and may deliver lipoprotein-derived cholesterol to follicle cells during androgen synthesis, thereby regulating women’s reproductive function. The second role of ApoE in ovarian tissue is the downregulation of androgens by theca cells to favor progesterone synthesis [31]. Oriá et al. [31] also reported that the increase of ApoE concentration in follicular fluid correlated with a decrease in fertility with age.

Von Wald et al. [28] investigated the involvement of specific apolipoproteins in the process of human oocyte maturation and age-related infertility, as molecular constituents of follicular fluid, and registered an increase in ApoE with age, which parallels the lower number of retrieved mature oocytes in older women. Follicular ApoE was present in diverse heterogeneous complexes including very-low-density lipoproteins (VLDL), intermediate-low-density lipoproteins (IDL), low-density lipoproteins (LDL), and high-density lipoproteins (HDL) that vary with patient age and differ from the blood plasma lipoprotein complexes [28]. The authors concluded that age-related variation in follicular ApoE content and distribution in the cholesterol particles may be associated with the decrease in production of mature oocytes and age-related decline in fertility potential [28].

The ApoEε4 allele at the ApoE locus, encoding ApoE, significantly increases risk of poor health, and it is present in many populations, at a relatively high percentage [32]. As ApoE is a major supplier of cholesterol, a precursor for the production of ovarian estrogen and progesterone, *ApoE* is taken into account as a potential candidate gene that may influence human reproductive potential. The results obtained by Jasienska et al. [32] support this hypothesis, showing that in regularly menstruating women those with genotypes with at least one ApoEε4 allele had significantly higher levels of mean luteal progesterone than women with genotypes without ApoEε4, which indicates higher fertility potential. The authors suggest that the higher level of progesterone in women with the ApoEε4 allele may be linked with an evolutionary mechanism maintaining the ancestral and health-worsening ApoEε4 allele in human populations [32]. Van Exel et al. [33] examined a rural Ghanaian population with a high pathogen exposure for selective advantages of ApoEε4 for survival and fertility. They found a nonsignificant, but positive survival benefit, adjusted for socioeconomic status, tribe and sex. Among women aged ≥40 years, ApoEε4 was not associated with the lifetime number of children. On the other hand, however, ApoEε4 was associated with higher fertility in women exposed to high pathogen levels. Women carrying one ApoEε4 allele had on average one more child, and those carrying two ApoEε4 alleles had 3.5 more children, in comparison to women who were not carrying an ApoEε4 allele. The authors concluded that, contrary to the case in affluent societies, ApoEε4 did not carry a survival disadvantage in this rural Ghanaian population [33]. Additionally, ApoEε4 promotes fertility in highly infectious environments. Its adverse associations in affluent modern societies with later onset diseases of aging further characterize ApoEε4 as an example of antagonistic pleiotropy [33]. Accumulating evidence indicates a dual effect of ApoEε4 during the lifespan, being beneficial to women’s fertility, but detrimental to late-life chronic diseases, only in settings of poor hygiene and sanitation [31]. The ApoE also could influence reproduction through involvement in the embryo development, as indicated by the reported association of the *ApoE* gene with trisomies 13, 18, and 21 [34]. Corbo et al. [35] investigated possible associations between ApoE genotype, past fertility, and Alzheimer disease onset age. ApoE genotypes were determined in a group of 176 women with sporadic AD, and the number of children each woman had delivered was recorded. The authors compared ApoE genotype distribution in parous and nulliparous women, and reported that the ApoE3/ApoE3 genotype is associated with higher fertility and the ApoE4-carrying genotypes with lower fertility [35]. When the influence of fertility and ApoE genotypes on AD onset age were analyzed, parity was found to be associated with a significantly lower AD onset age (73.8 ± 6.2 years) than nulliparity (80.7 ± 5.0 years; *p* = 0.0007) among subjects carrying ApoE3/ApoE3 and ApoE3/ApoE2 genotypes. On the contrary, there was no similar effect among ApoE4 carriers. Summarizing, it can be concluded that past fertility may have a relevant effect on AD onset age, an effect which is influenced by ApoE genotype [35].

In conclusion, the ApoE biological function, analyzed in the context of female fertility, is multifaceted. The correlation between increased ApoE concentration in follicular fluid and decreased fertility with age should be underlined. The follicular ApoE level and distribution in the cholesterol particles may be also associated with a decrease in the production of mature oocytes and an age-related decline in fertility potential. The role of ApoE in steroidogenesis, therefore regulating women’s reproductive function, is also worth noting. The ApoE genotype also influences the fertility potential of women. A higher level of progesterone was reported in women with ApoEε4 allele, which was also associated with higher fertility in women exposed to high pathogen levels, and promotes fertility in highly infectious environments. ApoEε4 during the overall lifespan may be beneficial to women’s fertility, but detrimental to late-life chronic diseases. Additionally, the reported association of the *ApoE* gene with trisomies 13, 18, and 21 showed that ApoE could also influence reproduction through involvement in embryo development. Interestingly, the dependencies between ApoE genotype and past female fertility may have a relevant effect on AD onset age.

#### 2.1.2. ApoE in Male Fertility

It is suspected that ApoE, as one of the lipid components of semen, plays an important role in the functional processes of sperm [36,37]. The study of the *ApoE* gene polymorphism conducted by Gerdes et al. [29] showed higher fertility potential in men with ε3ε3 genotypes compared to the ε3ε4 and ε2ε3 genotypes. Reports by Mahley et al. [38] and Setarehbadi et al. [37] documented the existence of relationships between the ε3ε4 genotype and decreased ApoE concentration, and authors hypothesized that a connection probably exists between decreased ApoE concentration and increased risk of male infertility. The possible impact of *ApoE* gene polymorphism on reproductive potential was examined by Corbo et al. [39], who investigated the distribution of ApoE genotypes and alleles, as well as blood plasma cholesterol level, in relation to number of children in 160 women and men of postreproductive age from southern Italy. As a control group, the population of reproductive age (<40 years) was also examined for ApoE allele frequencies and cholesterol levels, to verify whether the study sample of elderly people was representative of the fertile population [39]. Based on the results obtained, the authors concluded that the ɛ2 allele seems to be associated with the lowest reproductive efficiency, and the ɛ3 allele with the highest. The different total cholesterol levels associated with ApoE genotypes could influence steroidogenesis and as a consequence determine the observed differential fertility [39]. The aim of Paoli et al.’s [40] study was to look for correlations between ApoE polymorphism in humans and semen quality, in order to check whether ApoE genotypes have a significant effect on spermatogenesis. The 235 infertile men were found to have oligoasthenoteratozoospermia (OAT) and secretory azoospermia, with various accompanying andrological disorders such as varicocele, hypogonadism, cryptorchidism and orchiepididymitis. The control group was composed of 203 normozoospermic men, with no andrological disorders [40]. The authors reported that ɛ3/ɛ3 and ɛ3/ɛ4 alleles are not associated with the quality of spermatogenesis, as there was no significant difference between the normozoospermic and OAT groups or between the normozoospermic and the azoospermic groups for either of the genotypes examined. Given that men suffering from azoospermia are completely infertile, these results are of particular importance. If ApoE polymorphisms were correlated with male fertility, a comparison of normozoospermic and azoospermic subjects should demonstrate a significant difference in genotype distribution. However, no such difference was found [40]. It can therefore be inferred that ApoE genotypes have no effect on semen quality, and this demonstrates once again that the use of number of children as an index of fertility is not indicative of real male reproductive potential [40].

To summarize, ApoE, as one of the lipid components of semen, plays an important role in the functional processes of sperm. The differences in total cholesterol levels associated with ApoE genotypes could influence steroidogenesis and determine the observed differential fertility. Moreover, higher fertility potential in men with the ε3ε3 genotypes compared to the ε3ε4 and ε2ε3 genotypes, and associations between the ε3ε4 genotype and decreased ApoE concentration have been documented, which probably indicates a connection between decreased ApoE concentration and increased risk of male infertility. On the other hand, ɛ3/ɛ3 and ɛ3/ɛ4 alleles are not associated with the quality of spermatogenesis, and ApoE genotypes have no effect on semen quality or male reproductive potential.

### 2.2. ApoE in Gynaecological Diseases Influencing Fertility

#### 2.2.1. Endometriosis

Collazo et al. [41] examined whether gene polymorphisms in ApoE are associated with endometriosis and/or endometriosis-associated infertility. The authors conducted a cross-sectional genetic association study of women with surgically confirmed endometriosis and no surgical evidence of the disease. While they found no association between the ApoE genotype and diagnosis of endometriosis, the patients with endometriosis who reported at least one spontaneous pregnancy loss (SPL) were three times more likely to be ɛ2 carriers and twofold less likely to be ɛ4 carriers. Compared with ɛ3 carriers, patients with endometriosis who were ɛ2 carriers and had at least one live birth, reported four times the rate of spontaneous pregnancy loss, while ɛ4 carriers were <0.4-fold less likely to report an SPL. Collazo et al., 2012 [41] suggested that there may be an association between ApoE allelic frequency and spontaneous pregnancy loss in patients with endometriosis, which appears to be independent of mechanisms associated with infertility.

#### 2.2.2. Polycystic Ovary Syndrome

Polycystic ovary syndrome (PCOS) is associated with hyperinsulinemia and peripheral insulin resistance, both of which have been related to dyslipidemia [42]. Heinonen et al. [43] investigated the possible role of ApoE in the dyslipidemia seen in PCOS by determining the frequencies of ApoE alleles and genotypes, using the polymerase chain reaction (PCR) combined with restriction fragment length polymorphism (RFLP) analysis. The authors observed that the profiles of allele and genotype frequencies of ApoE confirm the equilibrium state between ApoE polymorphism and polycystic ovary syndrome, and suggest that ApoE does not play a major role in the development of hyperlipidemia in the group of women with polycystic ovary syndrome [43]. Fan et al. [44] in their study investigated ApoE-containing HDL-associated PAF-AH (HDL-PAF-AH) and total (ApoE-containing+ApoE-poor) HDL-PAF-AH activities in 291 patients with polycystic ovary syndrome and 281 control women. Patients with hyperandrogenism+oligo/anovulation+polycystic ovaries (PCO) or hyperandrogenism+PCO had lower total ApoE-containing and ApoE-poor HDL-PAF-AH activities, while those with oligo/anovulation+PCO showed decreased total and ApoE-poor HDL-PAF-AH activities, compared to the control women. Decreased total and ApoE-containing HDL-PAF-AH activities may contribute to the pathogenesis of PCOS and potentially link to related complications responsible for inflammation and oxidative stress [44]. Liu et al. [45] investigated the association between ApoE genotypes and the risk of PCOS, and evaluated the effects of the genotypes on metabolic profile and oxidative stress in women from south-west China. ApoE genotypes were determined by PCR and restriction fragment length polymorphism analysis. The authors reported that no significant differences exist in the frequencies of ApoE genotypes (E2/2, E2/3, E2/4, E3/3, E3/4, E4/4) and alleles (ε2, ε3, ε4) between PCOS and control groups. Compared with ε3 homozygotes (ApoE3/3), however, ε2 carriers (ApoE2/2+ApoE2/3+ApoE2/4) had significantly higher body mass index, waist circumference and waist-to-hip ratio, a more adverse glucose and insulin metabolic profile, lower high-density lipoprotein-cholesterol (HDL-C) and ApoA1 levels, higher triglyceride/HDL-C (TG/HDL-C) ratio and prevalence of metabolic syndrome, whereas ε4 carriers (ApoE3/4+ApoE4/4) had higher total cholesterol and low-density lipoprotein-cholesterol (LDL-C) levels in patients with PCOS [45]. Liu et al. [45] concluded that no significant associations were present between any ApoE genotype and PCOS, however, the ApoEε2 allele seems to be related to abdominal obesity, insulin resistance and metabolic syndrome in women with PCOS.

The research results presented above enable us to conclude that, while the ApoE genotype is not associated with the diagnosis of endometriosis, the patients with endometriosis and a minimum of one spontaneous pregnancy loss (SPL) were more likely to be ɛ2 carriers and less likely to be ɛ4 carriers, and an association may be also observed between ApoE allelic frequency and SPL in patients with endometriosis, which seems to be independent from infertility causes. Moreover, it was reported that in women with PCOS no significant associations exist between any ApoE genotype and PCOS, and ApoE does not play a major role in the development of hyperlipidemia but decreased ApoE-containing HDL-PAF-AH activities may contribute to the pathogenesis of PCOS linked with inflammation and oxidative stress. In women with PCOS the ApoEε2 allele seems to be additionally related to abdominal obesity, insulin resistance and metabolic syndrome.

**Table 1:** The association of ApoE expression with other gynecological and male reproductive tract diseases, and in the context of some other types of pathology.

Pathological Condition	Origin of the Tested Material	Observed Changes	References
**Female Gynecological Diseases**			
Breast cancer	human	an inverse association between ApoE expression and prognosis, stage and response to treatment	[46]
	SAGE databases	increased expression of ApoE	[47]
Choriocarcinoma	human choriocarcinoma cell line, JAR	it was suggested that ApoE, which promotes receptor-mediated lipoprotein uptake, is secreted by the trophoblast to facilitate uptake of maternal lipoproteins	[48]
Endometrial adenocarcinoma (ECa)	human	in the poorly differentiated adenocarcinomas, ApoE was overexpressed 13.1-fold and 9.7-fold when compared with well and moderately differentiated tumors, respectively no difference in ApoE expression between well and moderately differentiated adenocarcinomas was observed increased expression of ApoE might represent a late event in the progression of well-differentiated endometrioid endometrial adenocarcinoma to a poorly differentiated endometrioid endometrial adenocarcinoma	[49]
Endometrial hyperplasia (EH)	human	the frequency of the ApoEɛ2 allele (Cys158) was higher in patients with EH+ECa than in healthy controls only ApoEɛ2 allele might be associated with concurrent occurrence of EH and ECa	[50]
Ovarian cancer	ApoE knock-out mice	the loss of ApoE affected the remodeling of ECM and the changed composition of ECM stimulated the malignant progression increase in the expression of several proteins of intraperitoneal ECM in ApoE−/− mice ECM in the abdominal cavity of ApoE−/− mice displayed a remodeled phenotype, and this altered microenvironment promoted the malignant progression of ovarian cancer	[51]
	SAGE databases	overexpression of *ApoE* gene, increased expression of ApoE level	[47]
	cell culture model OVCAR3 cells	The expression of ApoE in most ovarian serous carcinomas the expression of ApoE was significantly more often observed in the high-grade compared with low-grade SOCs ApoE is necessary for the proliferation and survival of OVCAR3 cells nuclear ApoE expression positively correlate with a favorable prognosis for patients, however, only in pleural effusion, not in solid tumors ApoE expression is important for the survival and proliferation of ApoE-expressing ovarian cancer cells	[52]
**Male Reproductive Tract Disorders**			
Prostate cancer	SAGE database	increased expression of ApoE	[47]
	human	the ApoEε4 allele increases cholesterol production which has been identified as an important risk factor for prostate cancer	[53]
	prostate cancer cell lines	non-aggressive cell lines carried ApoE ε3/ε3 or ε3/ε4 alleles, while the aggressive cell lines carried the ApoE ε2/ε4 alleles	[54]
	human	ApoE variants were not associated with the risk of prostate cancer or aggressive disease	[55]
	human	ApoE E3/E3 genotype may be a potential risk factor for prostate cancer and the ε4 allele may be a risk-reducing factor for prostate cancer	[56]
	human	ApoE present in blood could potentially be a discriminating biomarker between benign prostate hyperplasia and prostate cancer	[57]
**Other Pathologies**			
Overall incidence of cancer	a mendelian randomization study and meta-analysis	no significant relationship with the ApoE genotype	[58,59]
Gastric cancer	human	an inverse association between ApoE expression and prognosis, stage and response to treatment	[60]
Non-small cell lung cancer	human	an inverse association between serum ApoE expression and prognosis, stage and response to treatment	[61]
Pancreatic, stomach and colon cancer	SAGE databases	increased expression of ApoE	[47]

ECM—extracellular matrix, SAGE—serial analysis of gene expression, SOC—serous ovarian carcinoma.

## 3. The Role of ApoE Glycosylation

**Figure 3:**
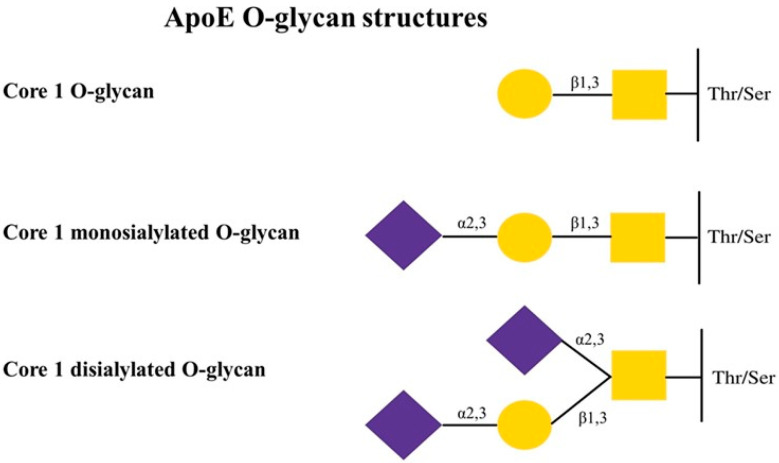
Typical O-glycan structures of human ApoE. 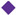 Neu5Ac—sialic acid (N-acetylneuraminic acid), 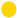 Gal—galactose, 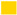 GalNAc—N-acetylgalactosamine, Ser—serine, Thr—threonine. Modification based on Flowers et al. [8].

Glycosylation is a post-translational modification of a protein, and as a result of this enzymatic reaction, carbohydrates are attached to the Asp or Thr/Ser of protein and a N- or O-glycosidic bond is formed, respectively [62]. ApoE is O-glycosylated and the glycosylation takes place during the transition through the Golgi and trans-Golgi network [63]. In this process, O-linked glycans like N-acetylgalactosamine (GalNAc) are attached to the exposed threonine or serine residues in the protein. ApoE has several possible glycosylation sites: in the hinge region (Thr194), within the hinge region (Ser197), in the N-terminus (Thr8 and Thr18) and also in the C-terminus (Thr289, Ser290 and Ser296) (see Figure 1) [7,64,65,66,67]. The intensity of ApoE glycosylation, e.g., sialylation, may vary significantly. This is due to the fact that ApoE can come from both tissues and cells. Additionally, these enzymatic processes are also influenced by the extracellular environment in which ApoE is present. An example of the occurring variability are the observed differences in the degree of ApoE glycosylation in blood plasma and in the cerebrospinal fluid. ApoE in blood plasma is less glycosylated than ApoE in CSF. It is related to reduced carbohydrate content in the molecule, which is probably due to the presence of unidentified glycosidases in the blood [8,68,69]. It was also noted that ApoE in CSF has a more strongly glycosylated C-terminal domain (CSF 37.8%, blood plasma 3.7%) and more abundant glycosylation in the hinge region (CSF 26.8% and 11.4% blood plasma), while ApoE in blood plasma is characterized by increased glycosylation in the N-terminal domain (CSF 0.2%, blood plasma 15.8%) [8]. The consequences of this seem to be differences in the binding of lipoproteins. Flowers et al. [8] reported that glycosylation of the ApoE C-terminal loop affects the preference for binding to lipoproteins. For example, the authors observed that the reduced glycosylation of this ApoE fragment in the blood plasma may allow for the connection of lipoproteins in various sizes and components to this domain [8,70]. The most frequent reports in the literature are on the role of ApoE expression in the formation and development of Alzheimer’s disease, also in the context of changes in the profile and degree of its glycosylation [71]. However, little is known about the biological role of ApoE glycosylation in the broadly understood aspect of human reproductive health, which is why we found this topic particularly interesting. The typical O-glycan structures of human ApoE are shown in Figure 3.

### 3.1. ApoE Glycosylation Changes in Preeclampsia

Preeclampsia is a group of disease symptoms that affects 2–7% of pregnancies. It is most often manifested by the presence of proteinuria and increased blood pressure, while in extreme cases it may be associated with the occurrence of liver and kidney disorders, coagulopathy or eclamptic seizures. These symptoms can lead to a variety of complications such as premature birth, abnormal fetal growth and increased maternal and fetal mortality, and may also affect the woman’s further life and health by a significant increase in the risk of cardiovascular disease [72,73,74,75]. Due to the complex pathophysiology and unclear etiology of preeclampsia, diagnostics are based on the observed clinical symptoms and the obtained results of laboratory tests, which reflect the body condition and the functioning of internal organs. However, there is currently no test that could be used in the diagnostics of this vascular pathology [76]. One of the possible ways to find a preeclampsia biomarker is the proteomic approach [77,78,79]. This conclusion was also reached by Atkinson et al. [80] who decided to check whether the serum/plasma may contain new biomarkers for preeclampsia. Using two-dimensional gel electrophoresis and difference gel electrophoresis, they compared serum/plasma from nulliparous women who had preeclampsia at 36–38 weeks of gestation, with healthy nulliparous women at a similar week of pregnancy. The serum/plasma used for the research was devoid of the six most abundant proteins. Then, using mass spectrometry and immunoblotting, they assessed the expression of selected proteins that showed significant differences in abundance during electrophoresis. In a subsequent study, the authors observed that the glycosylation pattern of ApoE in women with preeclampsia differs from that in healthy pregnant women [80]. In preeclampsia plasma, they found an increase in concentration of the deglycosylated ApoE isoform and a decreased level of glycosylated ApoE isoform. The authors suggested that ApoE in combination with other proteins can be used as a factor that will distinguish a healthy pregnancy from preeclampsia, and that ApoE deglycosylation can damage blood vessels by reducing HDL binding, which negatively affects the reverse transport of cholesterol from lipid-loaded macrophages, which in turn may connect preeclampsia and subsequent cardiovascular disease [80].

### 3.2. ApoE Glycosylation Changes in Breast Cancer

Breast cancer (BC) is one of the main cancers affecting women, with a noticeable tendency to occur more often in developed countries than in developing countries [81]. The main epidemiological factors that significantly increase the risk of this cancer include for example age, family history, oral contraceptives, oxidative stress, reproductive and hormonal factors, breast proliferative diseases, cancer, exposure to ionizing radiation, personal history of malignancy, a late decision about parenthood and the “Western lifestyle” [82,83,84,85]. Uen et al. [86] checked the relationship between the occurrence of post-translational ApoE modifications and the risk of breast cancer. The authors showed that blood plasma ApoE levels in BC are 1.07-fold lower, however they did not significantly differ between BC patients and healthy women. On the other hand, plasma ApoE levels significantly increased from stage I to stage III in BC (1.77-fold, *p* = 0.003), which was consistent with the study of Chen et al. [52]. Functional studies by Huang et al. [87] showed that glycosylated ApoE may be associated with the regulation of secretion, solubility, stability, and lipid binding. Nguyen et al. [88] reported that residues 261–299 in the ApoE C-terminal domain are critical for effective VLDL binding and ApoE self-association. It was documented that increasing VLDL secretion would diminish lipolysis and render VLDL clearance inefficient [89]. Lee et al. [65] observed that glycosylation of the T194 residue, located in the hinge region (residues 165–215), is an initial step following glycosylation of other sites on the C terminus of ApoE. Uen et al. [86] indicated that glycosylation of the S129 residue is 1.14-fold higher and is adjacent to the LDLR domain of ApoE (residues 130–150) [90]. It should be also mentioned that glycosylation of the S129 residue caused the electrostatic potential to be near zero, which might not interfere with the existing H-bond [91], and therefore the single slight increment in glycosylation observed might not influence the function of ApoE, although it is located between helixes 3 and 4 [92].

The variability of ApoE glycosylation observed in gynecological diseases such as preeclampsia and breast cancer clearly indicate that the degree of ApoE glycosylation may be also taken into account as a differentiating factor in some gynecological diseases. For example, an increase in concentration of the deglycosylated ApoE isoform and a decrease in the level of glycosylated ApoE isoform, in combination with other proteins, can be used as a factor distinguishing healthy pregnancy from preeclampsia. Although blood plasma ApoE levels in BC are lower than in healthy women, the differences were insignificant, but on the other hand plasma ApoE levels significantly increased from stage I to stage III in BC. As glycosylated ApoE may be associated with the regulation of secretion, solubility, stability, and lipid binding, any changes in its glycosylation may influence these processes. However, a single slight increment in glycosylation neutralizes electrostatic potential and thus inhibits interference with the existing H-bond and does not influence the biological function of ApoE.

## 4. Conclusions and Future Perspectives

The importance of ApoE expression, and its glycosylation changes in clinical practice, is still the subject of many studies. The role that ApoE plays in the mechanisms related to the maintenance of metabolism of fats in the human body is undeniable. Numerous studies have shown that both the expression of ApoE and the variability of its glycosylation are associated with disorders of the reproductive system in both women and men, and this may be an interesting research target. The variability of ApoE expression in the course of endometriosis, POCS, and other gynecological diseases such as breast cancer, choriocarcinoma, endometrial adenocarcinoma and hyperplasia or ovarian cancer, as well as the variability of its glycosylation observed in the course of diseases such as preeclampsia and breast cancer, only confirm that the biological function of ApoE is multidirectional, and the mechanisms of its action are not fully understood; hence it is worth taking a closer look at them also in terms of other diseases, including those related to impaired human fertility. The associations observed between ApoE expression, the type of its genotype and isoform, and male fertility disorders are also important, especially in the context of lipid metabolism disorders and their impact on male reproductive potential.

The degree of expression of ApoE glycans varies depending on where it occurs. For example, ApoE derived from the cerebrospinal fluid is richer in glycans than serum ApoE. The differences in ApoE glycosylation also concern the profile of the displayed glycoconjugates and the degree of their expression. In women the alterations in ApoE glycosylation were observed in the course of gynecological diseases such as preeclampsia or breast cancer, but such changes were also observed when ApoE glycosylation was analyzed in the context of female fertility problems. ApoE is involved in the physiological functions of the female gonads and the ApoE genotype could affect reproductive efficiency, taking into account that ApoE is also involved in implantation of the embryo in the uterus, and trans-placental transport of fat. Moreover, the changes in its expression are associated with oocyte maturation, age-related infertility or female hormone production. Alterations in ApoE glycosylation also play a role in male fertility. Sperm lipid components are important for their functional activity, participating in the process of male gamete capacitation in the fertilization process, and are also necessary for maintaining the proper vitality and maturation of sperm. It has been shown that in infertile men the lipid composition of the sperm membranes is changed compared to that of fertile men. ApoE plays an important role in the regulation of lipid metabolism and in their intercellular transport. However, little is known about the characteristics of ApoE glycosylation present in human seminal and blood serum/plasma, analyzed in relation to male reproductive health. A deeper analysis of ApoE glycosylation in the context of female and male fertility will enable us to broaden our knowledge of the biochemical and cellular mechanisms taking place with the participation of glycans, having a direct or indirect relationship with the fertilization process, and also provide us with the opportunity to contribute to the enrichment of the diagnostic panel in infertile women and men, which is particularly important for procedures involved in assisted reproductive techniques. Due to the fact that the above issues have not been clarified so far, they may constitute an interesting topic for researchers to explore. Understanding the mechanisms of glycoprotein glycosylation, including the role of a variety of enzymes in this process, and the interactions between glycans and their specific ligands related to the course of various diseases, including infertility, provides us with an opportunity to find a way to influence and modify the course of the glycosylation process, and thus to develop new therapeutic strategies.

## Data Availability

Not applicable.
